# Schwannoma of the eyelid: Apropos of two cases

**DOI:** 10.4103/0301-4738.53063

**Published:** 2009

**Authors:** Raja Anane Touzri, Khalil Errais, Rachida Zermani, Sarra Benjilani, Amel Ouertani

**Affiliations:** Department of Ophthalmology, Charles Nicolle University Hospital, Tunis, Tunisia; 1 Department of Hispathology, Charles Nicolle University Hospital, Tunis, Tunisia

**Keywords:** Benign tumor, eyelid, histopathology, schwannoma

## Abstract

Schwannoma, also referred to as neurilemmoma, is a benign tumor of peripheral nerve arising from Schwann cells that form the neural sheath. Schwannoma of ophthalmic interest is rare although it has been reported in relation with the orbit, and less frequently with the uveal tract and conjunctiva. Isolated eyelid schwannoma is extremely uncommon. Up until now, only eight cases have been reported in literature. Herein, we report two cases of eyelid schwannoma.

Schwannoma is a benign tumor arising from Schwann cells of peripheral nerves. The tumor is normally solitary, smooth-surfaced, slow-growing and generally asymptomatic. It may develop at any age and there is no gender predilection. Head and neck are one of the most frequent localizations.[1] Ocular tissues are rarely affected, occasionally schwannoma arises in the orbit, and infrequently in the uveal tract,[2] conjunctiva,[1] or sclera.[3] To our knowledge, only eight cases of eyelid schwannoma have been published in the literature so far.[4–9] We report here two cases of eyelid schwannoma.

## Case Reports

### Case 1

A 47-year-old man was referred to us with a one-year history of a slowly enlarging, painless nodule on his right lower eyelid. Ocular examination was normal except for the presence of a firm, non-tender nodule measuring 7 × 7 × 6mm in the medial third of the right lower eyelid. There were no clinical findings indicative of neurofibromatosis. The patient was operated under local anesthesia; tumor was completely removed via a horizontal eyelid crease incision. The postoperative course was uneventful and the patient was asymptomatic five years later.

**Figure 1:**
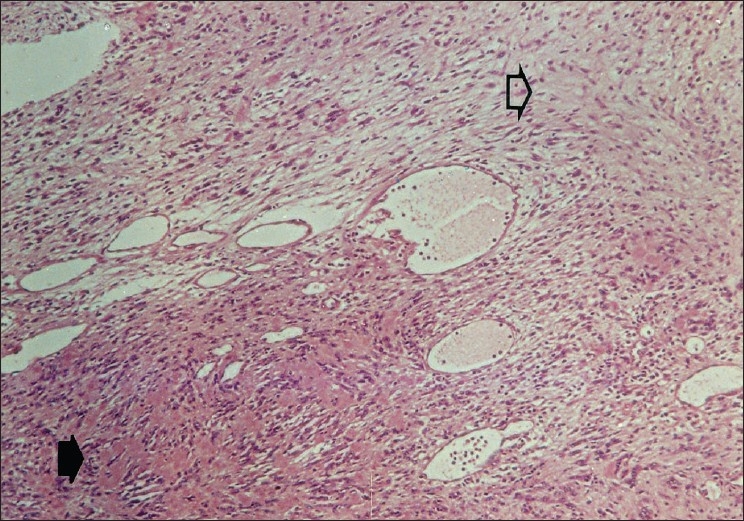
Histopathology of Case 1: admixture of compactly arranged interlacing fascicles, Antoni A pattern (black arrow), and loosely arranged cells separated by clear matrix, Antoni B pattern (white arrow) (H and E, ×25)

Pathological studies showed an encapsulated spheric mass (6mm in diameter) on macroscopic examination. Microscopic examination revealed a biphasic pattern, with highly cellular areas of non-pigmented spindle cells, containing elongated nuclei arranged in fascicles, which were separated by homogenous acellular material (Antoni A pattern). In other areas, the cells were more oval, and had rounded nuclei, clear cytoplasm and less basement membrane, and were loosely entwined within a clear myxoid matrix (Antoni B pattern) [Fig. 1]. No histopathologic features of malignancy were present. Immunochemistry for S-100 protein was strongly positive, but tumor cells did not react with HMB45. The final diagnosis was benign schwannoma Type A and B of the eyelid.

### Case 2

**Figure 2:**
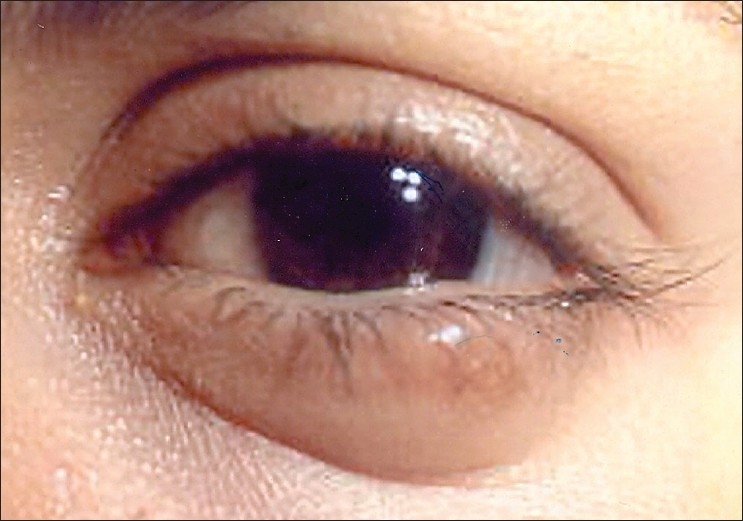
Clinical appearance of the left lower eyelid schwannoma of Case 2

A 20-year-old woman was referred to us for a chalazion of her left lower eyelid, which had been operated three times in four years. No tissues had been submitted for histopathological examination in earlier surgical procedures. Ocular examination was normal except for the presence of a firm, non-tender painless nodule measuring 13 × 8 × 8mm located in the middle of the left lower eyelid, independent of the lid margin [Fig. 2]. There were no clinical findings indicative of neurofibromatosis. The lesion was removed under local anesthesia by a full-thickness resection of the lid around the tumor.

On macroscopic examination the nodule was well-circumscribed, non-encapsulated, measuring 10mm in diameter with yellow appearance on cut sections. Microscopic examination revealed fascicules and bundles of non-pigmented spindle cells with elongated bland nuclei, and abundant extracellular collagen with adipocytes and squamous cells displaying an Antoni A pattern. There was no mitotic activity. Immunochemistry for S-100 protein was strongly positive, but tumor cells did not react with HMB45 [Fig. 4]. Final diagnosis was benign schwannoma of the eyelid.

## Discussion

**Figure 3:**
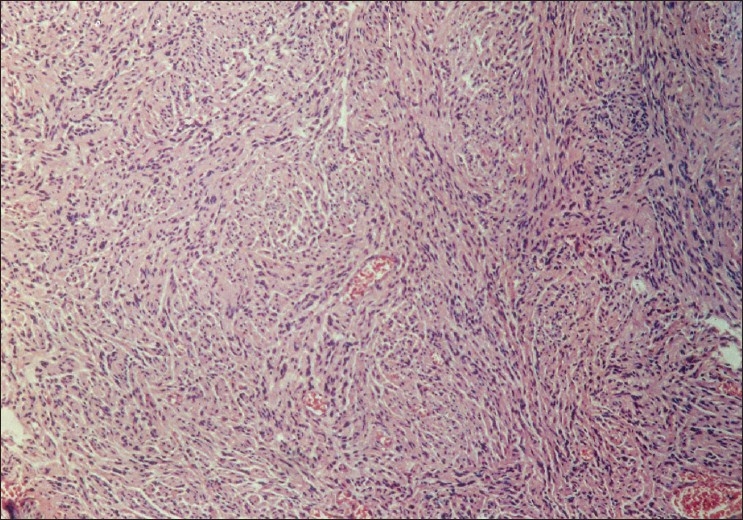
Histopathology of Case 2: Antoni A pattern with fascicules and bundles of non-pigmented spindle cells with elongated bland nuclei (H and E, ×25)

Schwannoma (or neurilemmoma) is made up of proliferating Schwann cells of peripheral nerve sheaths. It is a neoplasm which occurs wherever schwann cells are present, that is in any myelinated peripheral nerve. In most cases, while schwannoma is sporadically manifested as a single benign neoplasm, the presence of multiple schwannomas in one patient is usually indicative of neurofibromatosis 2 (NF-2) [Fig. 3]. However, the term schwannomatosis or neurilemmomatosis has been used to describe patients with multiple non-vestibular schwannomas with no other stigmatas of NF-2 or NF-1.[10]

**Figure 4:**
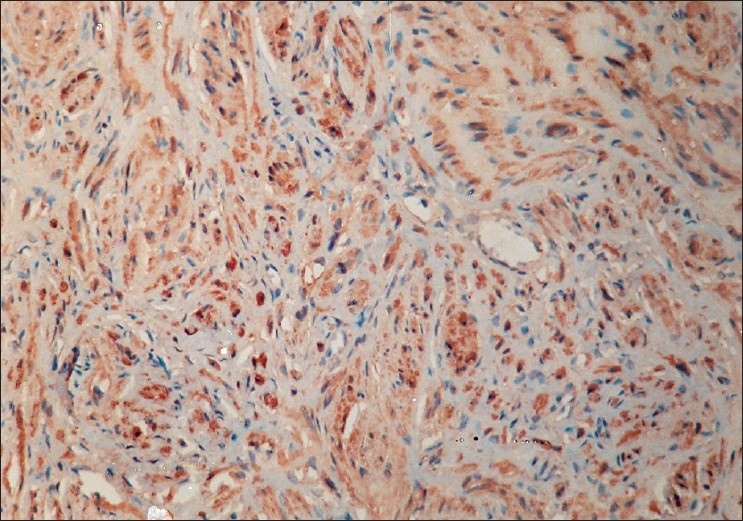
Immunochemistry of Case 2: Strong reactivity for S100 protein (H and E, ×25)

Our two patients had isolated eyelid schwannoma with no family history or clinical findings of NF-1 or NF-2. Schwannoma is well known to develop in the orbit, constituting 1% of the orbital tumors in a series by Rootman *et al.*[11] Isolated cases have been described in the conjunctiva,[1] the uveal tract,[2] and the sclera.[3] Its occurrence on the margin of the eyelid is extremely rare. In Pubmed database we only found eight published cases [Table 1]. In our department of ophthalmology and in a period of 10 years, schwannoma represents 0.7% (2/282) of the eyelid neoplasms. In the López-Tizón series also schwannoma was rare and represented only 0.1% (2/2400) of eyelid neoplasms[8] Age range of published cases of eyelid Schwannomas was between seven and 70 years [Table 1]. Clinically, the tumor is a solid, slowly progressive mass with no pain. Due to its rarity and unusual location, eyelid schwannoma is frequently clinically confused with other diagnoses like chalazion[5] (our Case 2), or inclusion cyst.[8] To avoid eventual recurrence, surgical excision is indicated and has to be complete.

**Table 1:** Summary of published cases of eyelid schwannomas and our two cases

Authors	Age (years)	Sex	Localization	Histological pattern	Number of cases
Reeh MJ[5]	60	F	LLE near MC	Antoni A and B	1
Baijal GC[4]	19	M	LLE near LC	Antoni A and B	1
Shields JA[5]	63	F	LLE near MC	Antoni A and B	1
Shields JA[6]	7	M	RUE	Antoni A	1
Siddiqui MA[7]	53	M	RUE	Antoni A and B	1
López-Tizón E[8]	41	F	RUE	Antoni A and B	2
	70	F	LLE		
Chung YR[9]	66	F	LUE	Antoni A and B	1
Our cases (2002)					
Case 1	47	M	RLE	Antoni A and B	2
Case 2	20	F	LLE	Antoni A	

F: female, M: male, RLE: right lower eyelid, RUE: right upper eyelid, LLE: left lower eyelid, LUE: left upper eyelid, MC: medial canthus, LC: lateral canthus

Pathologically, schwannomas classically show a mixture of two patterns, the Antoni Type A densely cellular pattern and the Antoni Type B edematous and disorganized pattern.

The most important feature for diagnosis remains the strong reactivity to S100 protein in immunochemistry.[1–7] Negativity of tumor cells for HMB45 rules out the diagnosis of melanotic lesion.[1]

The rare occurrence of eyelid schwannoma should be kept in mind in the differential diagnosis of any solid palpebral lesion, especially in case of recurrent chalazion.

When they are isolated, they are mostly benign. In neurofibromatosis they might rarely undergo malignant transformation.[1]
